# Transcriptome analysis of the typical freshwater rhodophytes *Sheathia arcuata* grown under different light intensities

**DOI:** 10.1371/journal.pone.0197729

**Published:** 2018-05-29

**Authors:** Fangru Nan, Jia Feng, Junping Lv, Qi Liu, Shulian Xie

**Affiliations:** School of Life Science, Shanxi University, Taiyuan, China; Chuo University, JAPAN

## Abstract

The Rhodophyta *Sheathia arcuata* is exclusively distributed in freshwater, constituting an important component in freshwater flora. This study presents the first transcriptome profiling of freshwater Rhodophyta taxa. A total of 161,483 assembled transcripts were identified, annotated and classified into different biological categories and pathways based on BLAST against diverse databases. Different gene expression patterns were caused principally by different irradiances considering the similar water conditions of the sampling site when the specimens were collected. Comparison results of gene expression levels under different irradiances revealed that photosynthesis-related pathways significantly up-regulated under the weak light. Molecular responses for improved photosynthetic activity include the transcripts corresponding to antenna proteins (LHCA1 and LHCA4), photosynthetic apparatus proteins (PSBU, PETB, PETC, PETH and beta and gamma subunits of ATPase) and metabolic enzymes in the carbon fixation. Along with photosynthesis, other metabolic activities were also regulated to optimize the growing and development of *S*. *arcuata* under appropriate sunlight. Protein-protein interactive networks revealed the most responsive up-expressed transcripts were ribosomal proteins. The *de-novo* transcriptome assembly of *S*. *arcuata* provides a foundation for further investigation on the molecular mechanism of photosynthesis and environmental adaption for freshwater Rhodophyta.

## Introduction

The Rhodophyta constitutes an ancient derived monophyletic eukaryotic lineage. As a member of archaeplastida, Rhodophyta originated from the primary photosynthetic endosymbiosis and subsequently spread plastid through secondary endosymbiosis to a diverse array of photosynthetic lineages [1, 2]. They are primarily marine in distribution, with less than 3% of the over 6500 species occurring in truly freshwater habitats [3, 4]. Though owning a relatively low diversity compared with the marine group, freshwater rhodophytes are usually important constituents of stream floras, either in terms of abundance or distribution from local scale to biomes [5]. Genus *Sheathia* is a typical freshwater Rhodophyta and inhabited exclusively in streams or rivers. It belongs to the Florideophyceae, growing as gelatinous gametophyte filaments, with beaded appearance, varying from blue-green, olive, violet, and gray to brownish. *Sheathia* can be found the year round but most abundant in late winter and spring, with the growing rate accelerating in December and decreasing in June throughout a year [6, 7]. Species of *Sheathia* are reported worldwide, and numerous species have been collected from different continents. *S*. *arcuata* is one of the most widespread species in the genus and has recently been reported from numerous localities [8].

Light is one of the important environmental factors regulating photosynthesis, growth and reproduction of photosynthetic organisms. Physiological responses to changing light intensity have been examined extensively [9, 10]. Variation of growth rate, pigment content and photosynthetic characteristics in response to irradiance have been investigated in freshwater red algae [10, 11]. However, little is currently known regarding the molecular mechanisms affecting the regulatory and biochemical pathways of freshwater red algae *Sheathia* in response to irradiance. Previous report has confirmed that *Sheathia* was typically shade-adapted plants, whereas some species can tolerate high irradiances and have mechanisms to avoid photo damage [10]. Thus, analyzing the gene expression patterns in response to different irradiance will provide a molecular basis for their environment adaption. Transcriptome analysis using next-generation sequencing is a powerful tool for examining complex molecular mechanisms. It provides a complete reference profile to understand genome content, gene function, gene expression under various conditions [12]. High-throughput RNA-sequencing (RNA-Seq) provides new perspectives for analyzing functional complexity of transcriptomes [13–15]. It has been used to analyze different gene expression patterns of different morphological types or under different conditions in higher plants [16, 17]. Whereas the transcription profiling is still unknown for freshwater Rhodophyta.

In this study we presented the transcriptome profile of the typical freshwater taxa *S*. *arcuata*, analyzed the coding gene contents and function annotations based on BLAST against multiple databases. The significantly different expressed genes under different irradiances were analyzed, thus laying a foundation for investigation on the molecular mechanism for the environmental adaption of freshwater Rhodophyta.

## Materials and methods

### Sample collection and preparation

Samples of *S*. *arcuata* were collected in Nanlaoquan, Jinci Park, Shanxi province, China (37°42′24.02″N; 112°26′31.76″E) on June 20th and December 22nd, 2015. The park where the samples were collected is open to public and no specific permissions are requested for field sampling, and we confirm that the field studies did not involve endangered or protected species. According to the statistical data of Monthly Averaged Clear Sky Insolation Incident On A Horizontal Surface in Taiyuan (https://eosweb.larc.nasa.gov/cgi-bin/sse/grid.cgi?email=zhenhuawan@gmail.com), the collection dates were selected when strongest and weakest light intensities occurred empirically. Both collection days were sunny and the light intensities were measured using a curing radiometer. Specimens of *Sheathia* growing at the same location with similar wet weight were collected at different dates. Other parameters related to the water conditions were measured with pH & EC waterproof (HANNA instruments, Woonsocket RI USA) when samples were collected, and the results were showed in Table 1. Physiochemical factors including temperature, pH, current velocity, total dissolved solids and electrical conductivity of the underground water in the sampling site were relative stable, except for the considerable different light intensities.

**Table 1:** Average water parameters over multiple samplings of the sampling site in this study.

Sampling date	strains	water temperature	pH	Current velocity	TDS (total dissolved solids)	EC (electrical conductivity)		light intensity (μmol photons/m^2^/s)
**June 20th, 2015**	12	16.5°C	6.23	13 cm/s	998		498	1462
**December 22nd, 2015**	12	16.0°C	6.23	13 cm/s	996		486	274

The thalli were washed using distilled water and frozen in liquid nitrogen as soon as possible after collection at the sampling site. Total RNA of each specimen was extracted according to Holmes and Bonner [18]. After the sample were treated with DNase, RNA degradation and contamination were monitored on 1% agarose gels. RNA purity was checked using the NanoPhotometer® spectrophotometer (IMPLEN, CA, USA). Concentration of RNA was quantified using Qubit (Thermo Fisher Scientific) and integrity of RNA was tested using Agilent 2100 (Agilent technology). RNA samples used for subsequent analyses were with values of A260/A280 ratios between 1.9 and 2.1, RNA 28S:18S ratios higher than 1.0, and RNA integrity numbers (RINs) ≥ 6.8. The extracted RNA samples of each group (high light intensity and low light intensity) were pooled from 3 individual thalli before subsequent handling.

### Library preparation for transcriptome sequencing

A total amount of 1.5 μg RNA per sample was used as input material for the RNA sample preparations. Sequencing libraries were generated using NEBNext® Ultra™ RNA Library Prep Kit for Illumina® (NEB, USA) following manufacturer’s recommendations and index codes were added to attribute sequences to each sample. mRNA was purified from total RNA using poly-T oligo-attached magnetic beads. Fragmentation was carried out using divalent cations under elevated temperature in NEBNext First Strand Synthesis Reaction Buffer (5X). First strand cDNA was synthesized using random hexamer primer and M-MuLV Reverse Transcriptase (RNase H). Second strand cDNA synthesis was subsequently performed using DNA polymerase I and RNase H. Remaining overhangs were converted into blunt ends via exonuclease/polymerase activities. After adenylation of 3’ ends of DNA fragments, NEBNext adaptor with hairpin loop structure were ligated to prepare for hybridization. In order to select cDNA fragments of preferentially 150~200 bp in length, the library fragments were purified with AMPure XP system (Beckman Coulter, Beverly, USA). Then 3 μl USER Enzyme (NEB, USA) was used with size-selected, adaptor-ligated cDNA at 37°C for 15 min followed by 5 min at 95°C before PCR. Then PCR was performed with Phusion High-Fidelity DNA polymerase, universal PCR primers and Index (X) Primer. At last, PCR products were purified (AMPure XP system) and library quality was assessed on the Agilent Bioanalyzer 2100 system. The clustering of the index-coded samples was performed on a cBot Cluster Generation System using TruSeq PE Cluster Kit v3-cBot-HS (Illumina) according to the manufacturer’s instructions. After cluster generation, the library preparations were sequenced on an Illumina Hiseq platform and paired-end reads were generated.

### Transcriptome analysis

Clean reads were produced by removing reads containing adapter, reads containing ploy-N and low quality reads from raw data. At the same time, Q20 (corresponding to sequencing quality with 99% accuracy rates) and GC-content of the clean data were calculated. All the downstream analyses were based on clean data with high quality. Transcriptome assembly was accomplished based on the clean data using Trinity [19] with min_kmer_cov set to 2 and all other parameters were set default. Gene function was annotated based on BLAST search against the following seven databases: Nr (NCBI non-redundant protein sequences); Nt (NCBI non-redundant nucleotide sequences), Swiss-Prot (a manually annotated and reviewed protein sequence database) with 10^−5^ e-value cutoff and KOG/COG (Clusters of Orthologous Groups of proteins) with 10^−3^ e-value cutoff. Automatic annotation ServerKO (KEGG Ortholog database) was conducted with 10^−10^ e-value cutoff. GO (Gene Ontology) annotation was conducted using Blast2GO v2.5 [20] with 10^−6^ e-value and customized script. Pfam (Protein family) annotation was based on hmmscan in the HMMER 3.0 package with e-value 0.01 [21, 22].

### Quantification of gene expression levels and differential expression analysis

Gene expression levels were estimated by RSEM [23] for each sample, with the clean data mapping back onto the assembled transcriptome and read count for each gene was obtained from the mapping results. Prior to differential gene expression analysis, the read counts were adjusted by edgeR program through one scaling normalized factor for each sequenced library, which was designed for datasets with no biological replicates [24]. Differential expression analysis of two samples was performed using the DEGseq package [25, 26]. P-value was adjusted using q-value [27]. q-value < 0.005 and |log_2_(foldchange)| > 1 were set as the threshold for significantly differential expression.

### Quantitative real-time PCR (qRT-PCR) validation

Total RNA extracted for library preparation were used as template of RT-PCR. Reverse transcribed cDNA were used to conduct quantitative real-time PCR with SYBR Green Dye (TakaRa SYBR Premix Ex Taq Ⅱ). Five genes from differentially expressed gene pools based on the bioinformatics analysis were selected including *psb*U, LHCA4, *pet*H, *pet*B and *pet*C. The translation initiation factor 5A (*elF5a*) was selected as internal control gene according to previous literature [28]. Amplification primers for selected genes were shown in S1 Table. The amplification procedures were 95°C for 30 s, 40 cycles of 95°C for 5 s, 53°C for 30 s, followed by dissolution stage of 95°C for 15 s, 53°C for 1 min and 95°C for 15 s. Specificity of the qPCR products was estimated based on melting curve. Expression values of each gene were calculated using the method proposed by Pfaffl [29]. Results of qPCR and RNA-seq data for the selected genes were compared.

### KEGG pathway enrichment analysis and Protein-protein interactive network construction

To understand the different functional pathways between the two samples, we used KOBAS software to test the statistical enrichment of differential expressed genes in KEGG pathways [30, 31]. The sequences of the differently expressed genes (DEGs) were BLAST to the genome of *Cyanidioschyzon merolae*, which was available of the protein-protein interaction (PPI) in the STRING database (http://string-db.org/) to get the predicted PPI of these DEGs. BLAST settings for constructing interaction networks were evalue = 1e-10 and max_target_seqs = 1. Then the PPI of these DEGs were visualized in Cytoscape [32].

## Results

### Transcriptome sequencing and assembly

**Fig 1:**
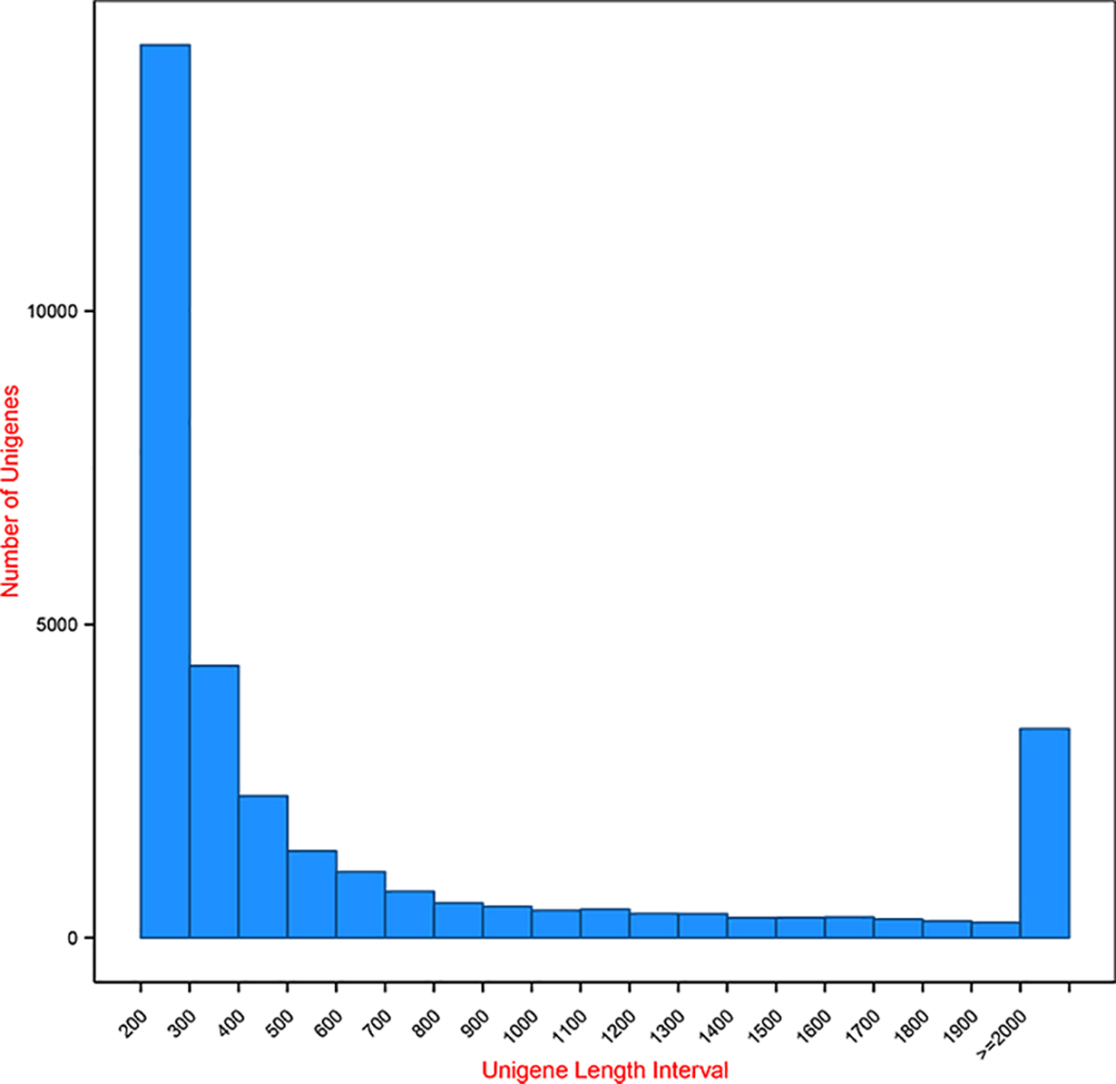
Annotated transcript-bar from clean reads of *S*. *arcuata*.

Two cDNA libraries prepared from samples collected at different seasons were sequenced using the Illumina Hiseq 2000 platform, producing database of 3.9 and 4.5 gigabyte respectively. Raw reads were 27.50 and 37.61 million paired-end reads for the algal sample respectively. These reads were 125 bp in length with high quality after reads filtering. After quality control approximately 26.66 and 34.84 million clean reads were obtained with similar GC content (Table 2). The sequence reads generated in this study have been deposited in GenBank under the accession numbers of PRJNA421565, PRJNA421415, PRJNA421429 and PRJNA421431. Mixed assembly of the clean reads generated 161,483 transcripts with the most abundant length interval of 200–300 bp (Fig 1). Transcripts with lengths ranging from 200–500 bp, 500–1000 bp, 1000–2000 bp and ≥ 2000 bp accounted for 87.71% (141,633), 7.62% (12,292), 2.31% (3,732), and 2.37% (3,826) of the total transcripts respectively.

**Table 2:** Statistics of RNA-sequencing quality of *S*. *arcuata* samples under different light intensities.

Sample	Raw reads	Clean reads	Clean bases (bp)	Error (%)	Q20 (%)	Q30 (%)	GC content (%)
**low light**	27499326	26661934	3.33G	0.01	97.49	94.19	54.98
**high light**	37608294	34837166	4.35G	0.01	97.26	93.95	55.70

### Gene annotations

**Fig 2:**
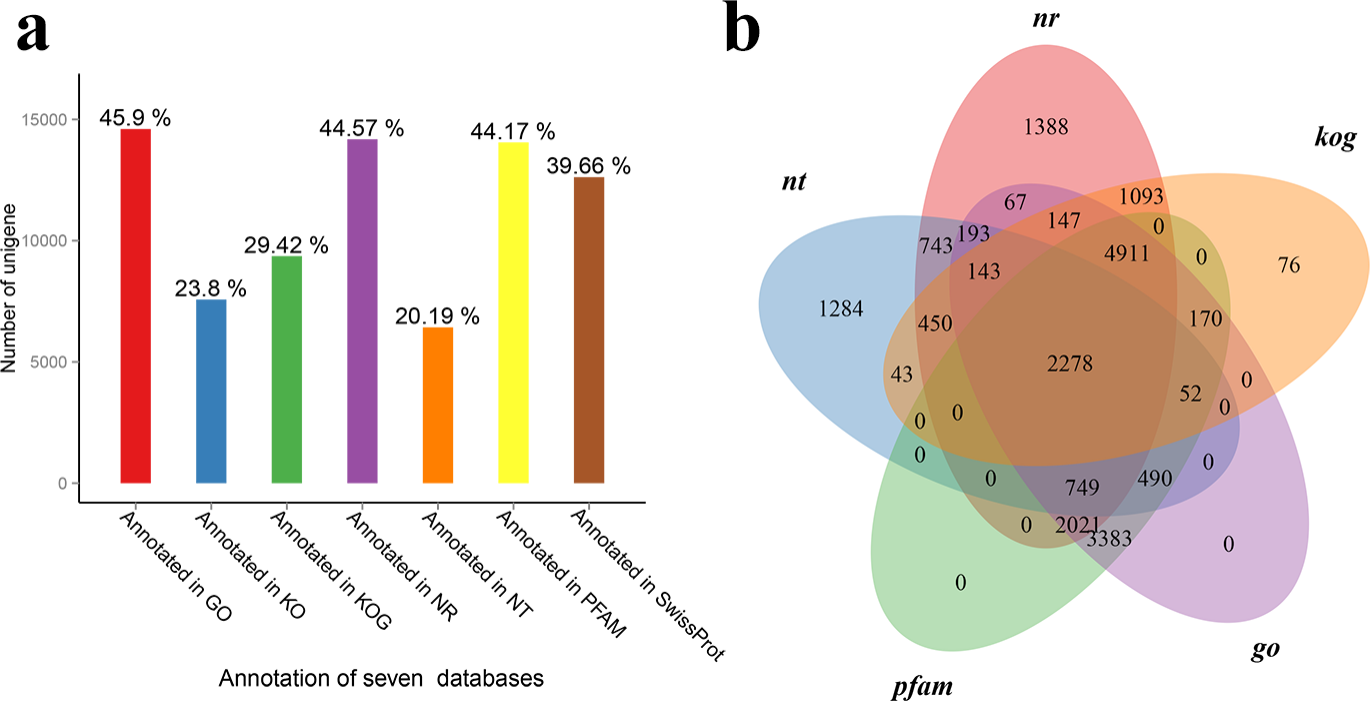
Function annotations of transcripts of *S*. *arcuata* based on BLAST against diverse databases. a. Numbers of transcripts annotated in seven databases; b. Venn diagram of transcripts annotated in five databases.

**Fig 3:**
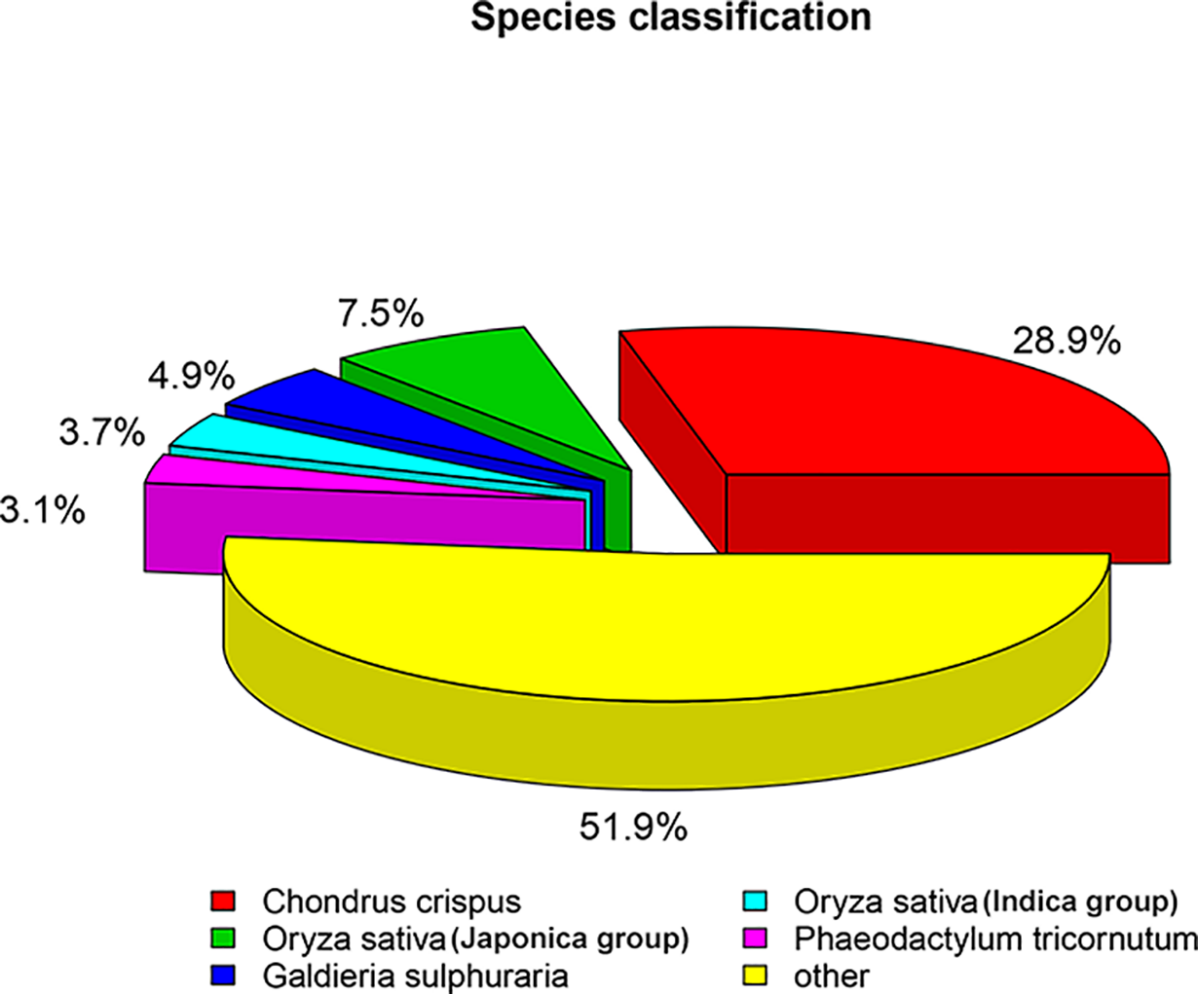
Classification of species with gene homology to *S*. *arcuata* based on Nr annotation result.

All 161,483 assembled transcripts were queried against seven curated databases (Fig 2A). Databases including Nr, Nt, KOG, GO and Pfam were selected to illustrate annotation venn diagram (Fig 2B). 2278 common genes were shared in the five annotation databases. Based on the Nr annotation result (Fig 3), the species with most homologous genes with *Sheathia* was the *Chondrus crispus* (marine Rhodophyta), followed by *Oryza sativa* (green plant), *Galdieria sulphuraria* (thermophilic Rhodophyta) and *Phaeodactylum tricornutumoth* (Bacillariophyta).

**Fig 4:**
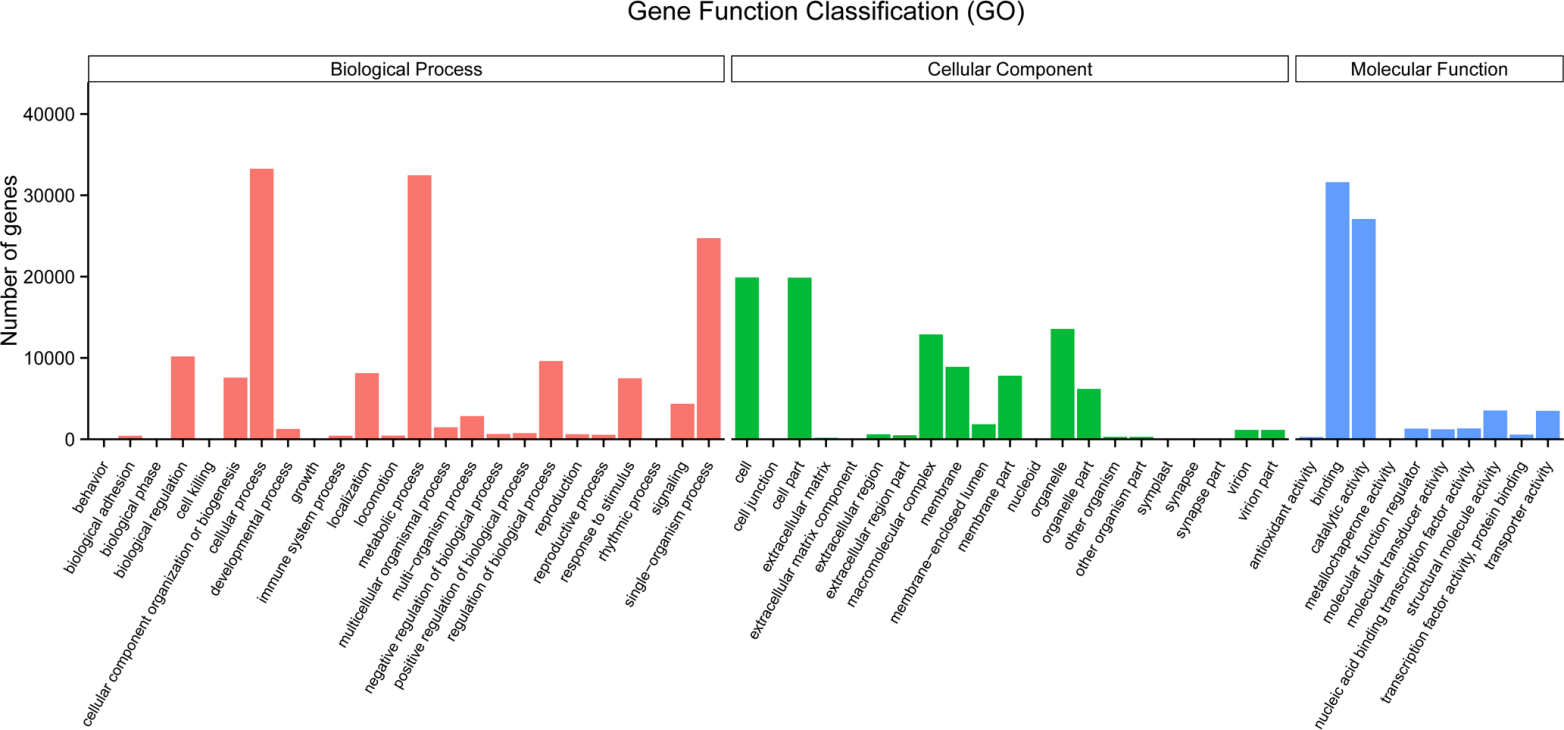
Gene function classification according to GO assignments for the predicted *S*. *arcuata* transcripts.

**Fig 5:**
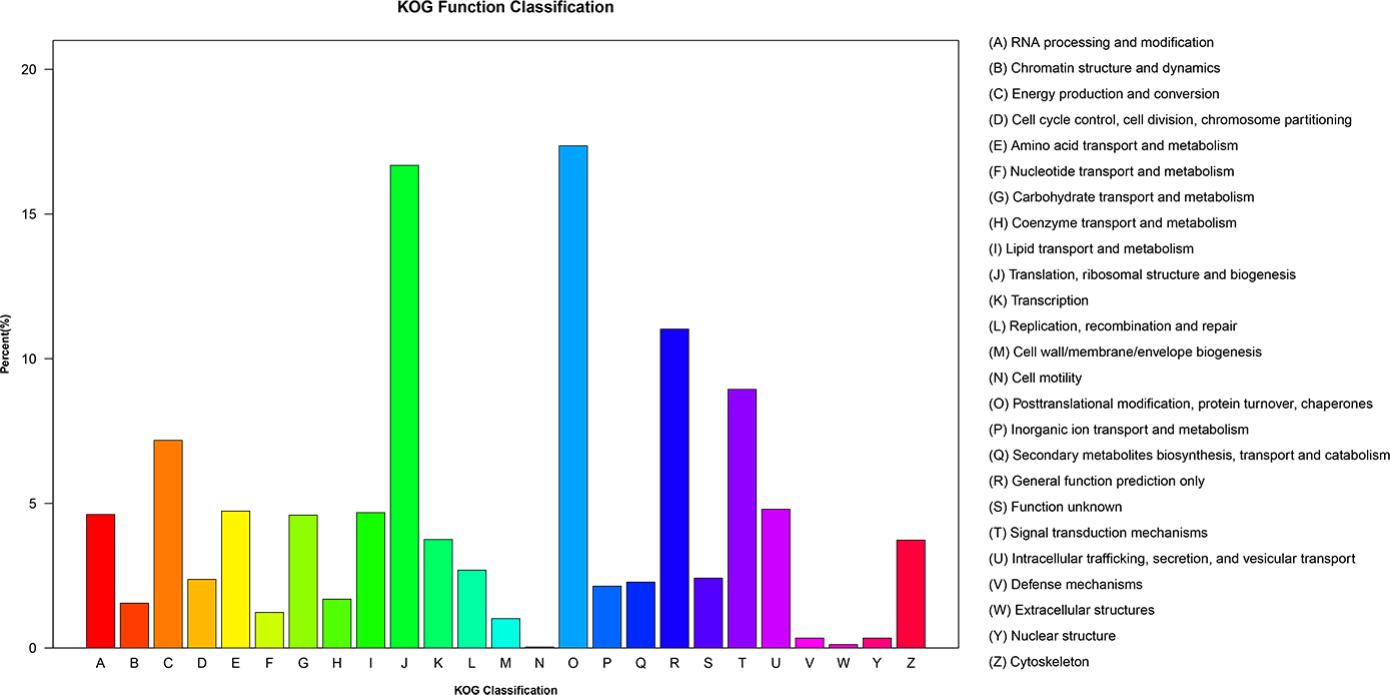
Gene function classification based on the KOG annotation for the predicted *S*. *arcuata* transcripts.

**Fig 6:**
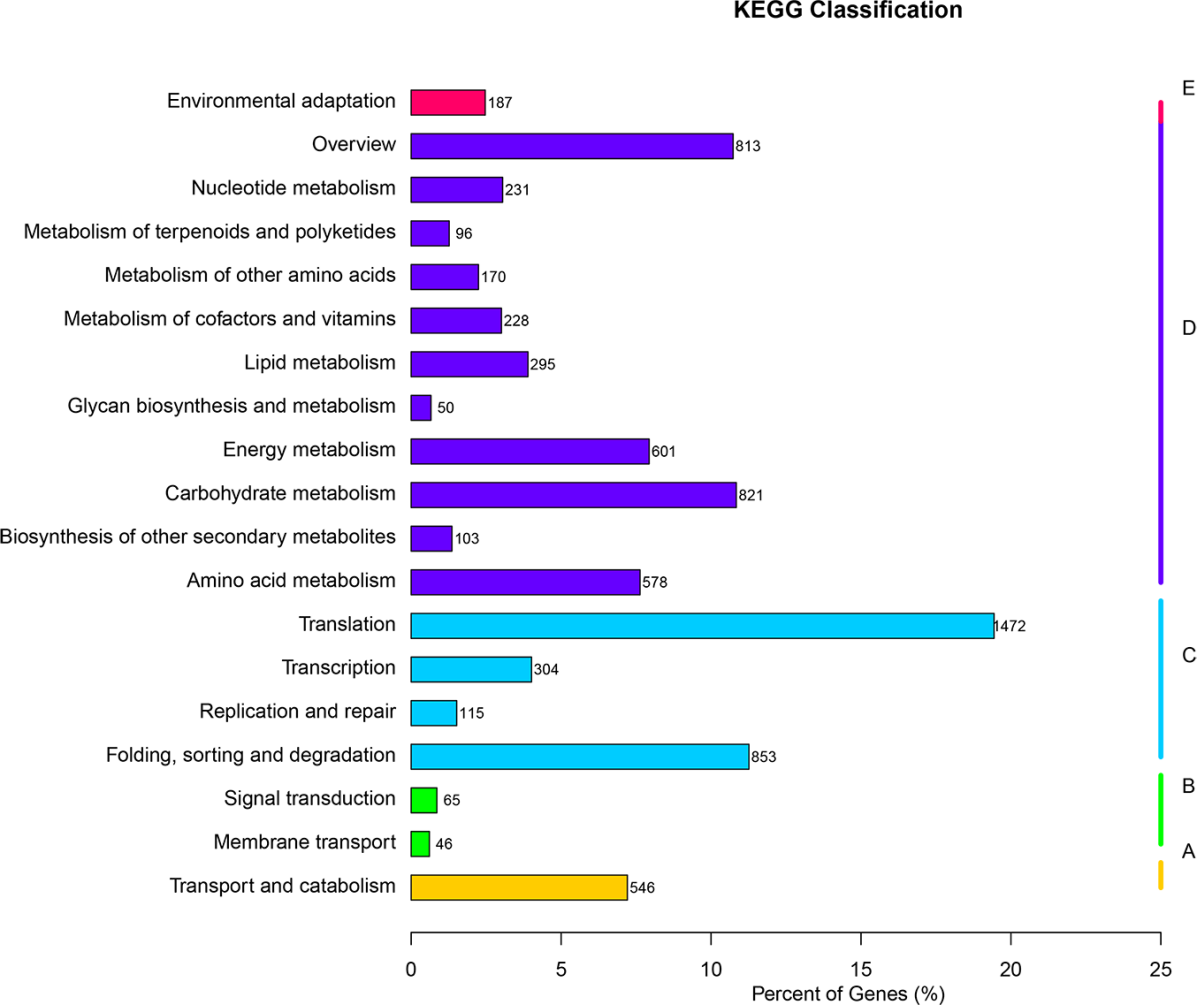
The biological pathways in *S*. *arcuata* according to the Kyoto Encyclopedia of genes and genomes (KEGG) database. A represents cellular processes; B represents environmental information processing; C represents genetic information; D represents metabolism; E represents organismal systems. Numbers on the right margin of each bar represents numbers of transcripts in the corresponding subcategories.

The predicted *S*. *arcuata* transcripts were classified according to GO assignments [33]. A total of 62,008 genes (38.4%) were assigned at least one GO term (Fig 4), among which 147,479 were assigned in the biological process category (Level 1), 70,427 in the molecular function category (Level 1) and 95,177 in the cellular component category (Level 1). These transcripts were further classified into functional subcategories. Genes corresponding to the ‘‘biological process” group (Level 1) were divided into 24 subcategories, among which “cellular process” (Level 2) comprised 22.6% and was the largest term. Genes corresponding to the ‘‘molecular function” group (Level 1) were divided into 10 subcategories, among which “binding” (Level 2) comprised 44.9% and was the largest term. Genes corresponding to the ‘‘cellular component” group (Level 1) were divided into 21 subcategories, among which “cell” (Level 2) comprised 20.9% as the largest term. Based on the KOG annotation result (Fig 5), 40,556 genes belonging to 25 categories were yielded. Among these categories, the largest group was genes for “Posttranslational modification, protein turnover, chaperones” cluster, owning 6,407 (15.8% of the totally annotated transcripts) in number. The biological pathways in *S*. *arcuata* were identified according to the Kyoto Encyclopedia of Genes and Genomes (KEGG) database (Fig 6). A total of 31,330 transcripts were mapped to 19 KEGG pathways in 5 categories (S2 Table). Among the 5 categories, the pathways represented by most transcripts were metabolism (17,224, 54.98% of the totally annotated transcripts), followed by genetic information (10,893, 34.77% of the totally annotated transcripts) and cellular processes 2285 (7.29% of the totally annotated transcripts).

### Differential gene expression analysis

**Fig 7:**
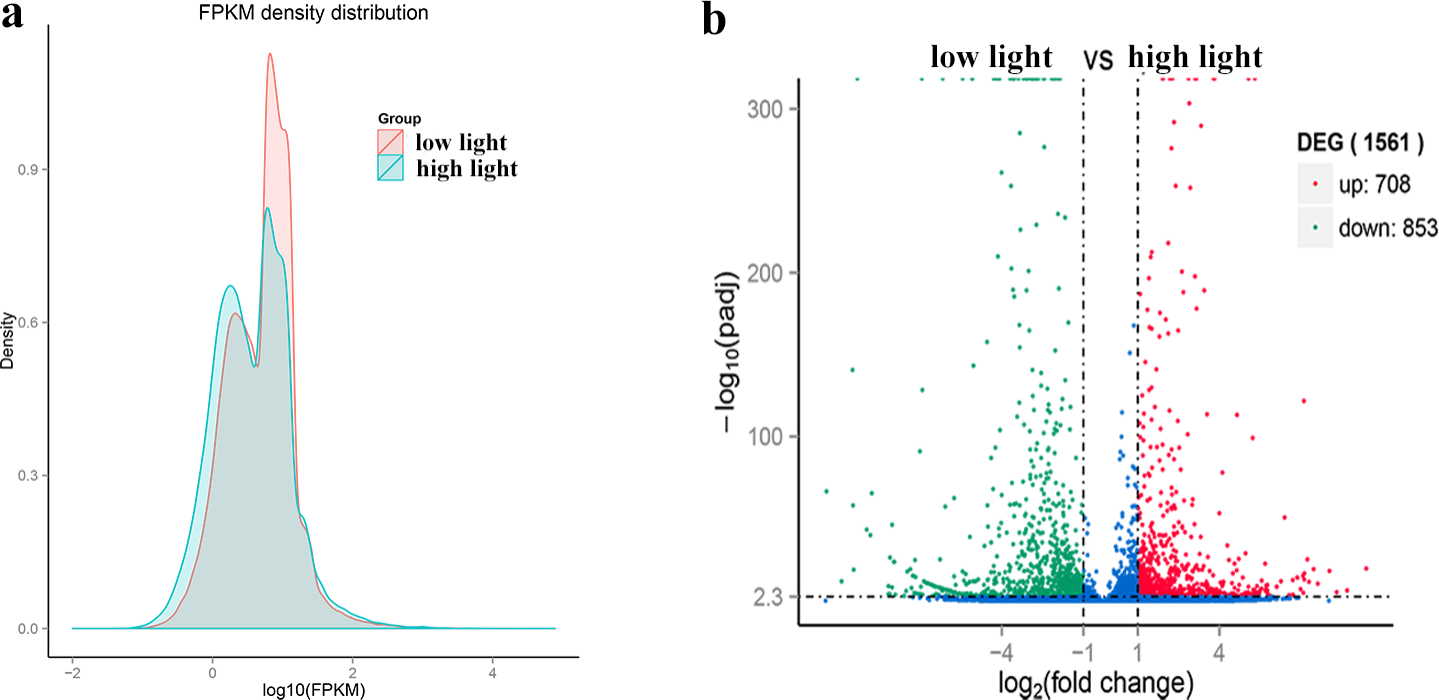
Different gene expression patterns for *S*. *arcuata* specimens collected at low and high light intensities. a. FPKM density distribution for *S*. *arcuata* specimens collected at low and high light intensities; b. Volcanoplot showing the up and down regulated genes for *S*. *arcuata* specimens collected at low and high light intensities.

Gene expression levels of each sample were counted using Trinity, with different gene expression patterns observed in the two *S*. *arcuata* samples illustrated in Fig 7. Considering the similar water conditions of the sampling site when the specimens were collected in this study, the different gene expression was caused principally by variant irradiance. Genes with the same expression levels owned different densities in the two samples, revealing variance of gene expression responding to light intensity (Fig 7A). Differentially expressed genes with statistically significance were observed with up-regulated and down-regulated genes mainly in the sample under high and low irradiance respectively (Fig 7B). Gene lists of down-regulated and up-regulated were listed in S3 and S4 Tables.

**Fig 8:**
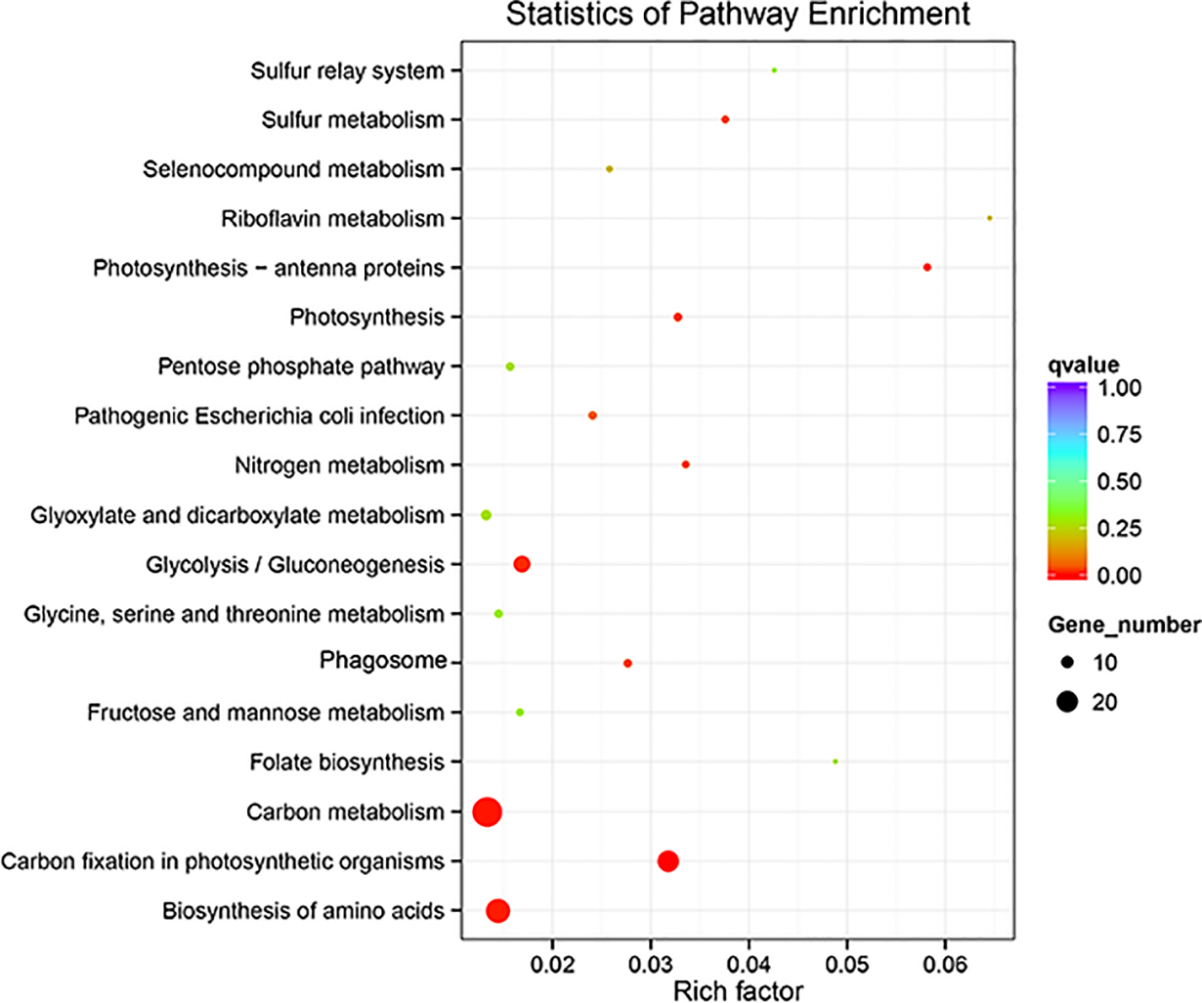
Enriched KEGG pathways for up-regulated genes in *S*. *arcuata* specimens collected under low light.

The top 18 enriched KEGG pathways involving up-regulated genes under low irradiance were illustrated as Fig 8. The enriched pathways of down-regulated genes in *S*. *arcuata* specimen under low irradiance were not statistically significant, with all the q-values larger than 0.5. Therefore they are not discussed in this study (S5 Table). On the other hand, the enriched pathways corresponding up-regulated genes are all statistically significant, with all the q-values evidently smaller than 0.5 (Table 3). The top 18 significantly up-regulated genes under low irradiance were involved in important metabolism pathways including energy metabolism (photosynthesis, photosynthesis-antenna proteins, carbon fixation in photosynthetic organisms, sulfur metabolism, nitrogen metabolism), carbohydrate metabolism (glycolysis/gluconeogenesis, pentose phosphate pathway, glyoxylate and dicarboxylate metabolism, fructose and mannose metabolism), amino acid metabolism (glycine, serine and threonine metabolism), overview metabolism (carbon metabolism, biosynthesis of amino acids), metabolism of other amino acids (selenocompound metabolism), metabolism of cofactors and vitamins (riboflavin metabolism, folate biosynthesis), cellular processes (phagosome) and genetic information processing (sulfur relay system).

**Table 3:** The top 18 enriched pathways in *S*. *arcuata* sample under low light.

Pathway term	Rich factor	q-value	Gene number
Carbon fixation in photosynthetic organisms	0.031796502	4.37e-07	20
Carbon metabolism	0.01335175	0.00433149	29
Biosynthesis of amino acids	0.014420063	0.005744689	23
Photosynthesis—antenna proteins	0.058139535	0.008377125	5
Glycolysis/Gluconeogenesis	0.016872891	0.011253209	15
Photosynthesis	0.032786885	0.024956933	6
Sulfur metabolism	0.037593985	0.031901757	5
Phagosome	0.02764977	0.040114472	6
Nitrogen metabolism	0.033557047	0.040114472	5
Gap junction	0.024096386	0.066983265	6
Selenocompound metabolism	0.025806452	0.197482769	4
Riboflavin metabolism	0.064516129	0.200305359	2
Folate biosynthesis	0.048780488	0.27698983	2
Pentose phosphate pathway	0.015665796	0.27698983	6
Glyoxylate and dicarboxylate metabolism	0.013245033	0.27698983	8
Sulfur relay system	0.042553191	0.295376788	2
Fructose and mannose metabolism	0.016666667	0.295376788	5
Glycine, serine and threonine metabolism	0.014527845	0.29597947	6

**Fig 9:**
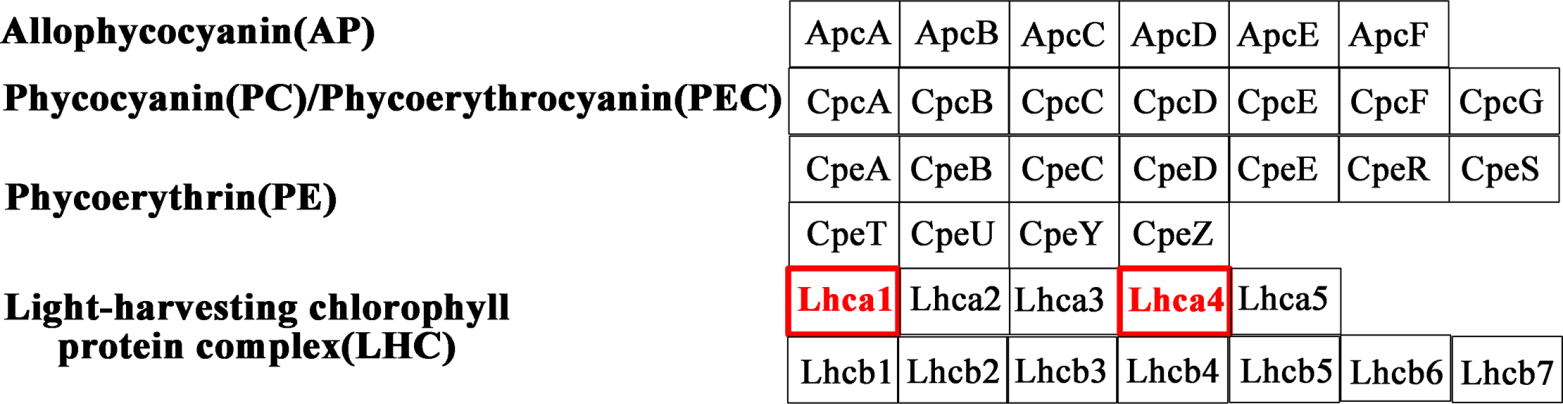
Up-regulated photosynthesis–antenna transcripts in *S*. *arcuata* specimen under low light. The transcripts in red were significantly up-regulated.

**Fig 10:**
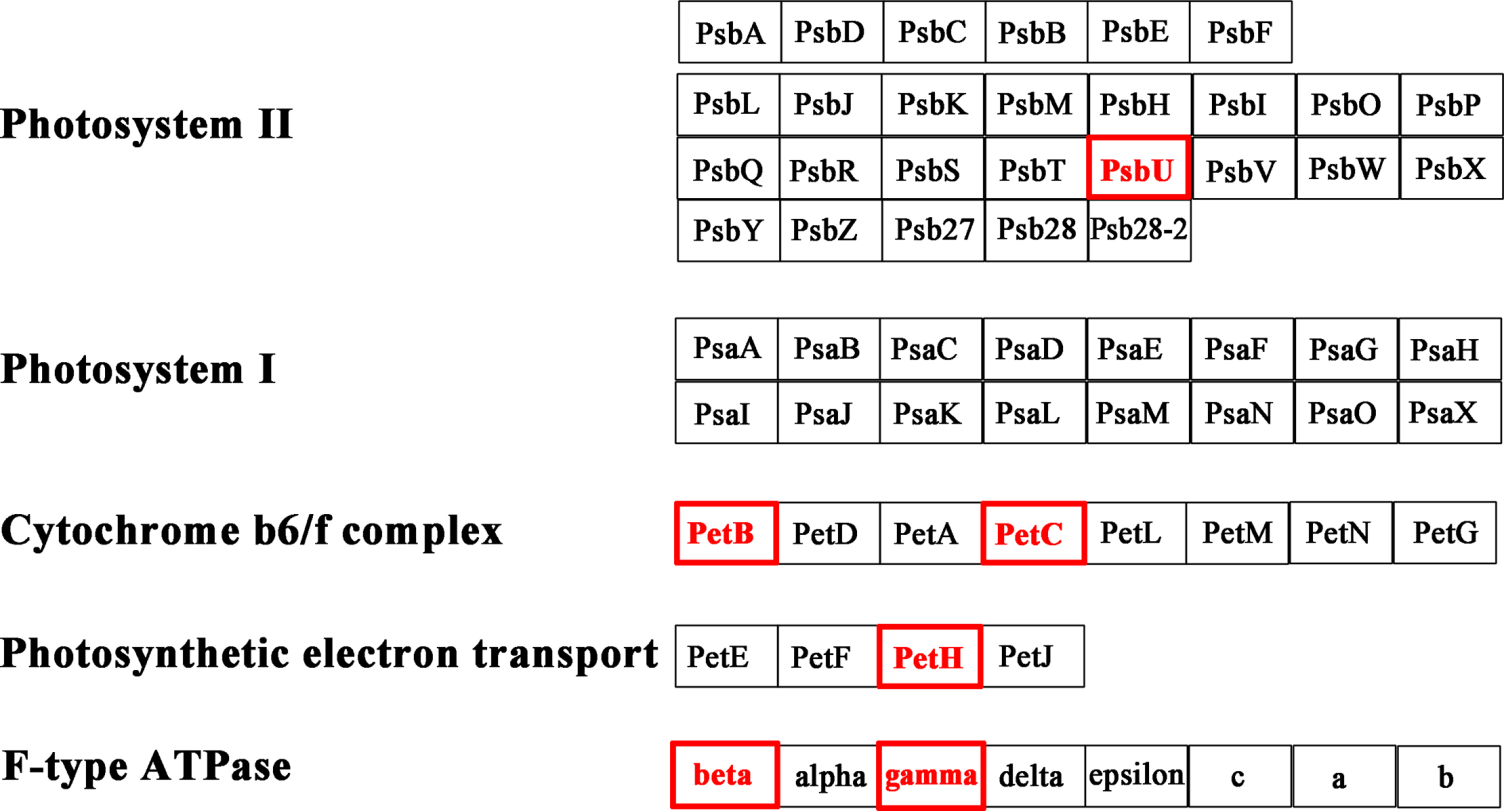
Up-regulated photosynthesis transcripts in *S*. *arcuata* specimen under low light. The transcripts in red were significantly up-regulated.

**Fig 11:**
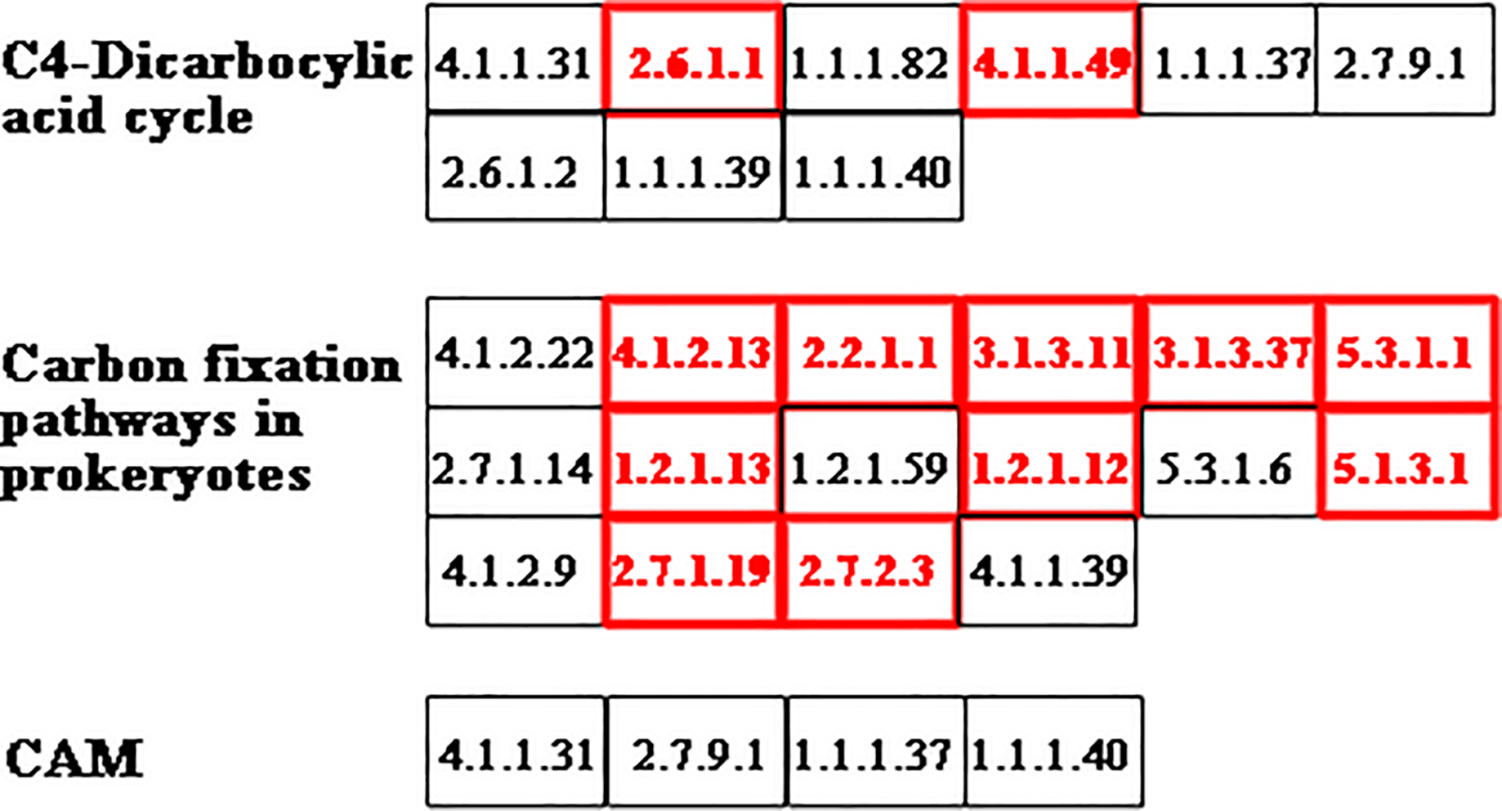
Up-regulated carbon fixation transcripts in *S*. *arcuata* specimen under low light. The transcripts in red were significantly up-regulated.

The significantly up-regulated transcripts involved in photosynthesis related pathways in *S*. *arcuata* specimen under low irradiance were showed in Figs 9–11 [30, 31]. Among photosynthesis—antenna proteins, significantly up-regulated transcripts were the light-harvesting chlorophyll complex LHCA1 and LHCA4, which were associated with the photosystem I (Fig 9). For photosynthesis apparatus, evidently up-expressed transcripts include PSBU in the photosystem II, PETB and PETC in cytochrome b6/f complex, PETH in photosynthetic electron transport, and the beta and gamma subunits of F-type ATPase (Fig 10). Moreover, the ATPase, which constituted part of the photosystem, were also up-expressed as a result of increased light absorption and electron transport. As the last step of photosynthesis, pathway of carbon fixation in photosynthetic organisms was the most enriched (Fig 11). The up-expressed transcripts involved in carbon fixation were as followed, phosphoenolpyruvate carboxykinase (EC 4.1.1.49), aspartate transaminase (EC 2.6.1.1), phosphoribulokinase (EC 2.7.1.19), transketolase (EC 2.2.1.1), sedoheptulose-bisphosphatase (EC 3.1.3.37), fructose-bisphosphate aldolase (EC 4.1.2.13), fructose-bisphosphatase (EC 3.1.3.11), Phosphotriose isomerase (EC 5.3.1.1), glyceraldehyde-3-phosphate dehydrogenase (NADP+) (EC 1.2.1.13), glyceraldehyde-3-phosphate dehydrogenase (EC 1.2.1.12) and phosphoglycerate kinase (EC 2.7.2.3).

**Fig 12:**
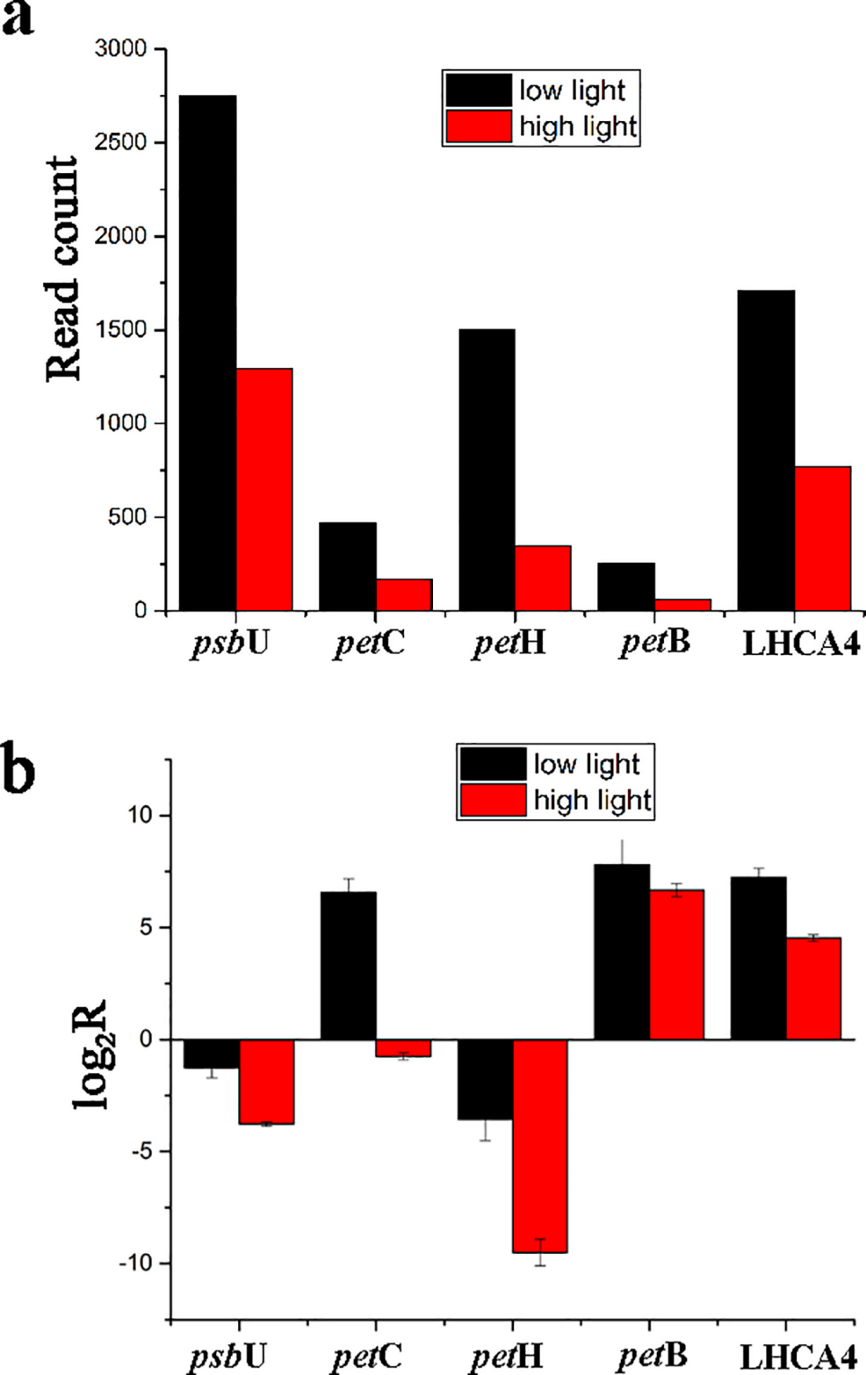
The qRT-PCR validation of focused genes in specimens collected at high and low light. a. Read counts of selected genes in each specimen based on high-throughput sequencing data; b. The qRT-PCR analysis of selected gene expression data.

Up-expression of focused genes in *S*. *arcuata* specimen under low light were validated by qRT-PCR, with the results showed in Fig 12. And the differential expression pattern revealed by qRT-PCR of selected genes was consistent with the high-throughput sequencing results, thus enhancing the statistical reliability based on sequencing data.

### Transcription factors

Transcription factors in *S*. *arcuata* (S6 Table) were identified and classified into different families using the iTAK pipeline (http://bioinfo.bti.cornell.edu/tool/itak) [34]. Results showed most abundant transcription factors involved in *S*. *arcuata* transcription process were regulatory genes including C2H2, C3H and orphan family. Both positive and negative transcriptional regulation of transcription factors by light has been documented. Different members in the families of common transcriptional factors and elongation factors were regulated diversely in the transcriptome profile of *S*. *arcuata* under weak irradiance (S3 and S4 Tables).

### Protein-protein interactions

**Fig 13:**
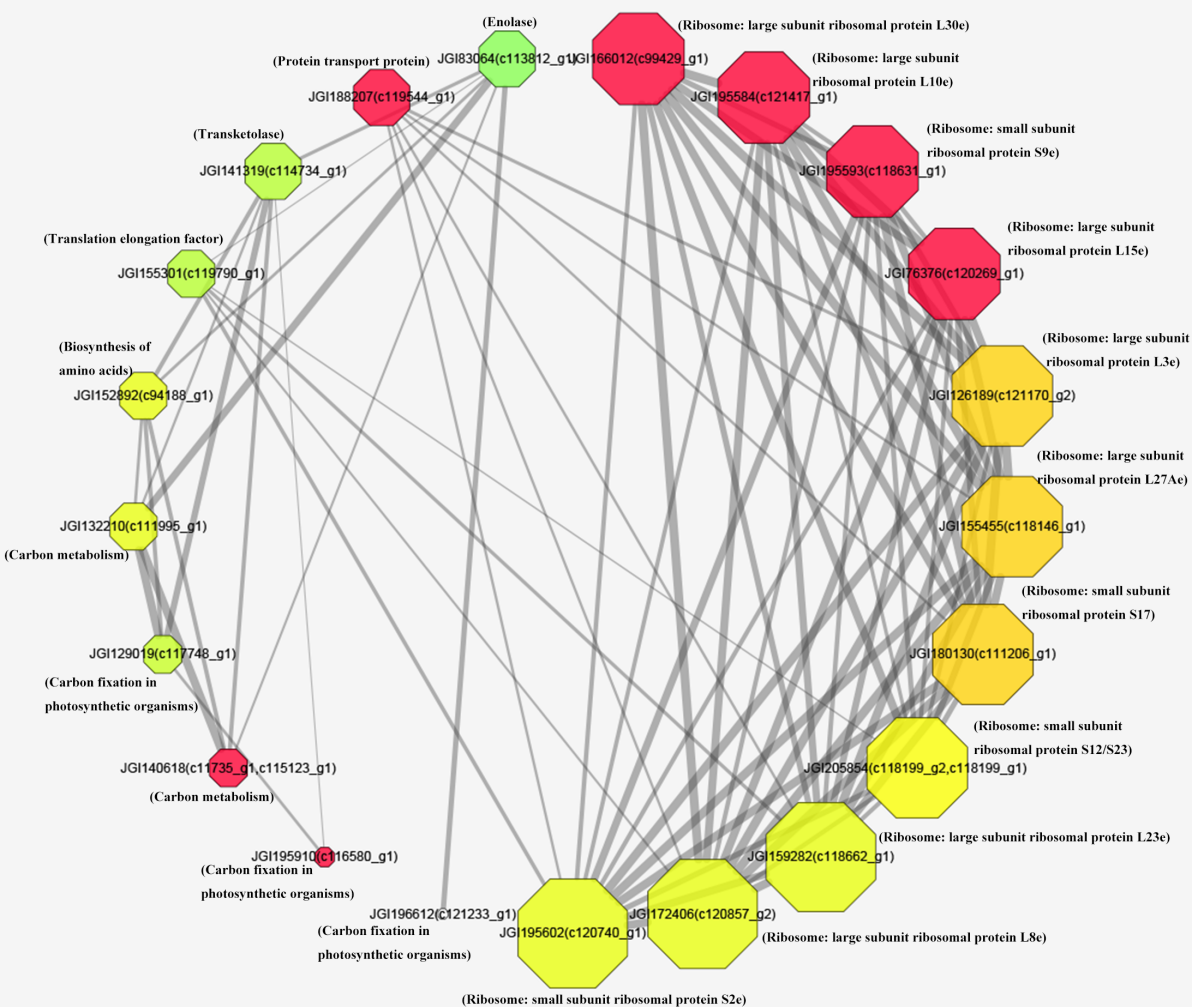
Protein-protein interactive network showing the relationship among up-regulated genes for *S*. *arcuata* specimen under low light. The size of each node represents interactive degree, with larger size corresponding to higher degree; the color of each node represents clustering coefficient, with colors ranging from green to red corresponding to lower to higher coefficients.

Interactive networks involving the up-regulated transcripts of *S*. *arcuata* in response to weak light intensity were shown in Fig 13. The results revealed that the transcripts in response to light were all cross-linked and in a closely-related network. The nodes with highest degrees were transcripts corresponding ribosomal proteins, followed carbon metabolism, protein transport proteins, translation elongation factors, biosynthesis of amino acids and carbon fixation proteins in photosynthetic organisms.

## Discussion

Despite the widely application of transcriptome sequencing in marine Rhodophyta [28, 35–39], transcriptome data have not been reported for freshwater red algae. This study presents the first transcriptome profiling of *S*. *arcuata*, which will enrich the repertoire of transcripts of the freshwater rhodophytes and provide more data for the further investigation on this plant lineage. Compared with the marine red algal samples with transcriptomes reported to date (*P*. *yezoensis*with 18,640 annotated transcripts [28]. Assembled transcripts of freshwater taxa *S*. *arcuata* was considerably larger. The overrepresented transcripts length obtained in this study is consistent with sequencing result of *P*. *yezoensis*, with the most abundant length distributed between 200–500 bp, while the GC content of the transcriptome in *S*. *arcuata* is lower than *P*. *yezoensis* [28].

Specimens used in this study were collected at the irradiance of 1462 and 274 μmol photons/m^2^/s respectively, which were similar with light intensities used in previous research of genus *Sheathia* by Necchi. Necchi proved the maximal photosynthetic rate (8.1 ± 0.5) occurred in specimens collected at the irradiance of 320 μmol photons/m^2^/s and the minimal rate (4.9 ± 0.6) was under 1510 μmol photons/m^2^/s based on oxygen evolution test [10]. Photosynthetic changing trend revealed by physiological parameters measured in Necchi’s research was consistent with the transcriptome regulation observed in our study.

Transcriptome analysis of *S*. *arcuata* grown under different light intensities in our study also shed light on the molecular mechanisms underlying the shade-adaption of this taxon. For *S*. *arcuata* specimen under weak light intensity, the up-expressed photosynthesis–antenna transcripts (LHCA1 and LHCA4, as observed in this study) facilitated more light absorption and thus improving the photosynthetic activity. Light-harvesting complexes (LHCs) associated with both photosystems I and II (in green lineage) and phycobilisomes (in cyanobacteria) served as the primary light-harvesting antenna for photosynthesis [1, 40]. LHCs are important constituents that facilitate photosynthetic function in response to light quantity and quality [41]. Moreover it was found LHCs responded more evidently than the phycobilisome in *S*. *arcuata* when grown under low light intensity, revealing the improved adaptive ability to surrounding environment and the advanced stage of LHCs in Rhodophyta evolution. It was consistent with previous report that in red algae, the photosynthetic apparatus represented a transitional state between cyanobacteria and chloroplasts of green lineage, with enhanced light-harvesting capacity by owning LHCs (light harvesting complexes) associated with PSI [42]. Another pathway in regulation of adaptive response to weak light was photosynthesis. The up-expressed transcripts including PSBU, PETB, PETC, PETH, the beta and gamma subunits of F-type ATPase contribute to the adaptive response. It was reported that in cyanobacteria and red algae, the PS-II system gene *psb*U encodes protein constituting part of the oxygen-evolving complex (OEC), which was also involved in stabilizing the oxygen-evolving machinery of PSII against high-temperature stress [43]. PETB participated in electron transferring in the photosynthesis, and PETC in the cytochrome b6/f complex was involved in mediating electron transfer between photosystem II (PSII) and photosystem I (PSI) [44]. In combination with the photosynthetic electron transport protein PETH, the transcripts related to protein network involved in the electron transport of photosynthesis were all up-expressed in *S*. *arcuata* under weak irradiance. Photosynthetic control of electron transport was a fundamental concept in the regulation of photosynthesis [45]. Additionally, regulatory pathway of *S*. *aucuata* in respond to weak light was carbon fixation. Phosphoenolpyruvate carboxylase (EC 4.1.1.49) was up-regulated in response to weak light in freshwater *S*. *arcuata*, combined with other enzymes to improve the carbon fixation activity. In marine macroalgae, phosphoenolpyruvate carboxylase (EC 4.1.1.49) was characterized as the only enzyme for dark carbon fixation [46]. The up-regulated transcripts of photosynthesis-antenna and photosynthesis apparatus triggered the increasing rate of carbon assimilation, thus fueling the growth of *Sheathia* specimen under the low light intensity. In contrast with previous report on higher plant including *Bryophyllum fedtschenkoi*, maize and barley, expression of phosphoenolpyruvate carboxykinase (EC 4.1.1.49) was down-regulated in response to decreased light [47]. It is speculated that the up-regulation of this enzyme under weak light in freshwater Rhodophyta *S*. *arcuata* is relevant to their shade-adaption. Our study revealed that for freshwater *S*. *arcuata*, transcripts involved in light harvesting, photosystem II, cytochrome b6 complex, photosynthetic electron transport and ATPase were all up-regulated thus enabling the increased photosynthetic function, which in turn provided sufficient energy and nutrients for fast growing of the plant under weak irradiance, which was consistent with the proposal that freshwater red algae were shade-adapted eukaryotic lineage [10].

Light provided fuel for photosynthetic electron transport and CO_2_ fixation. As the primary determinant of ATP levels and carbon metabolites, it served to modulate cellular processes based on complex transcriptional networks [48]. Genus *Sheathia* regulated relative small amount of photosynthetic genes under different light intensities compared with marine diatom *Chaetoceros neogracile*, which exhibited altered expression of most photosynthesis genes (48 out of 70) in response to high light according to previous report [49]. It maybe explain the molecular mechanisms for weak ability in environmental adaption of *Sheathia*, leading to the current situation of strict habitat demand and limited distribution of freshwater Rhodophyta globally.

Along with the increased photosynthesis, other metabolic pathways with up-regulated transcripts were also observed including carbon metabolism, biosynthesis of amino acids, glycolysis / gluconeogenesis, sulfur metabolism, phagosome and nitrogen metabolism. *S*. *arcuata* displayed sophisticated responses to optimize their photosynthesis and growth under weak light conditions. This finding was in line with previous research on marine red algae and diatom, which indicated that light regulated many important cellular processes, physiological processes and biochemical pathways [37, 50–51]. Among the diverse responsive transcripts, those corresponding to ribosomal proteins and involved in protein synthesis, proved highly-regulated for *S*. *arcuata* under weak light. In previous research on marine Rhodophyta *Chondrus crispus*, stress treatments caused decreased expression of protein synthesis-related genes [38], which implied indirectly that low light intensity was more appropriate for growth and development of *S*. *arcuata*.

Both up- and down-regulation of diverse transcription factors in *S*. *arcuata* in responding to different irradiances revealed the complexity of regulation network. Transcription factors have been enriched in early light-responsive genes according to recent genomic studies [52]. The transcription factors identified in this study can direct adaptive changes in gene expression of freshwater Rhodophyta in response to environmental light signals in further study.

## Conclusions

We present the first transcriptome profiling of freshwater Rhodophyta by conducting high-throughput RNA sequencing on *S*. *arcuata*. A total of 161,483 assembled transcripts were identified and different gene expression patterns under different irradiances were observed. The results revealed that photosynthesis-related pathways significantly up-regulated under the weak light, revealing the shade-adaption of freshwater red algae *S*. *arcuata*. Molecular mechanisms underlying shade-adaption are increased expression of transcripts corresponding to antenna proteins (LHCA1 and LHCA4), photosynthetic apparatus proteins (PSBU, PETB, PETC, PETH and beta and gamma subunits of ATPase) and metabolic enzymes in the carbon fixation. The most responsive up-expressed transcripts were ribosomal proteins in *S*. *arcuata* grown under low light intensity. The *de-novo* transcriptome assembly of *S*. *arcuata* laid the foundation for further investigation on environmental adaption of freshwater Rhodophyta.

## Supporting information

S1 Table Specific primers of each gene used for qRT-PCR experiments. (DOCX)

S2 Table KEGG_classification of the annotated transcripts in *Sheathia arcuata*. (XLSX)

S3 Table Down-regulated gene lists of *Sheathia arcuata* sample under low light intensity. (XLSX)

S4 Table Up-regulated gene lists of *Sheathia arcuata* sample under low light intensity. (XLSX)

S5 Table The top 20 enriched pathways of down-regulated genes in *Sheathia arcuata* specimen under low irradiance. (XLSX)

S6 Table Transcription factors identified in *Sheathia arcuata* and the related classification. (XLSX)
