# Supplementary material for: Transcriptome analysis of the typical freshwater rhodophytes Sheathia arcuata grown under different light intensities
Source: PLoS One. 2018 May 29;13(5):e0197729. doi: 10.1371/journal.pone.0197729 (PMC5973588; doi:10.1371/journal.pone.0197729)
Supplement: S1 Table — (DOCX) [file pone.0197729.s001.docx]

S1 Table Amplification primers used for qPCR of each gene

| gene | forward primer | reversed primer |
| --- | --- | --- |
| *psb*U | 5’-ATTCAACCTATGTGGCGGGG-3’ | 5’-TCCAGTCGCAATGTGAGCAT-3’ |
| *lhca*4 | 5’-AAGTGCTGTATGGACAGGGC-3’ | 5’-GAGATTGGCGGCTCTATGCT-3’ |
| *pet*H | 5’-AAGGTCGTCTACAACGAGCG-3’ | 5’-ATGGAGTAGAGGCGGACCTT-3’ |
| *pet*B | 5’-CGCTGGTCAGCGAGTATGAT-3’ | 5’-CGGTACACCCGTGACAATCT-3’ |
| *pet*C | 5’-ATCTCAGCTGCTTGACGCTT-3’ | 5’-CTGGAAAGGATGCGATTGCG-3’ |
| *elF5a* | 5’-TCAGATCGTACTTGCGCACT-3’ | 5’-GGCTCGTTGTATGAGCGTAGA-3’ |
